# Night Attire

**Published:** 1888-02-25

**Authors:** Lewis R. S. Tomalin


### NIGHT ATTIRE.

YOUR recommendation, in your issue of February 11th, of cotton or linen for night attire, in preference to wool, greatly interests me, as I have only lately translated and edited Dr. Jaeger's popular writings (*Health Culture.* Waterlow and Sons, 1s.), in which he strenuously insists on the superior hygienic value of wool. Your chief argument is that the skin requires a rest from the stimulus imparted to it in the day time by woollen underclothing; but, assuming that a rest is necessary, is not this sufficiently provided for by the absence of friction when the body is in repose at night? Wool, as I understand it, does not stimulate *per se*, and its friction on the skin is not noticeable so soon as it is habitually worn. On the other hand, animal wool has three great advantages over plant fibre (linen and cotton):—1. Animal wool is pervious to the passage of the vapour which the skin exhales. 2. Animal wool is non-conductive of the body's natural heat, and does not chill the skin, even when damp. 3. Animal wool does not, like plant fibre, from its nature retain the noxious malodorous emanations from the skin, and become itself malodorous. There has by this time been a very large and varied experience of sleeping in woollen clothing and bedding, and, so far as I have heard, the verdict in favour of wool is unanimous. In hot weather the body can throw off its *surplus* heat through the porous wool: in cold weather the non-heat-conductivity of wool defends the body's possession of its *natural* heat; in both seasons wool effectually protects the body from chill. LEWIS R. S. TOMALIN.

[There are undoubtedly many people to whom the constant wearing of flannel is a necessity; but for those in good health there is a sense of refreshment in putting off the flannel which was needed during the activities of the day, and putting on the fresh cotton or linen garment which is quite sufficient when the body is liable to no sudden change of temperature. Flannel encourages the action of the sebaceous glands even when it does not irritate the skin.—ED. *T. H.*]

